# Community science participants gain environmental awareness and contribute high quality data but improvements are needed: insights from Bumble Bee Watch

**DOI:** 10.7717/peerj.9141

**Published:** 2020-05-12

**Authors:** Victoria J. MacPhail, Shelby D. Gibson, Sheila R. Colla

**Affiliations:** 1 Faculty of Environmental Studies, York University, Toronto, Ontario, Canada; 2 Department of Biology, York University, Toronto, Ontario, Canada

**Keywords:** Community science, Citizen science, Bumble bees, Environmental awareness, Public participation in science, Program evaluation, Survey, Natural history, Pollinators

## Abstract

Bumble Bee Watch is a community science program where participants submit photos of bumble bees from across Canada and the United States for expert verification. The data can be used to help better understand bumble bee biology and aid in their conservation. Yet for community science programs like this to be successful and sustainable, it is important to understand the participant demographics, what motivates them, and the outcomes of their participation, as well as areas that are working well or could be improved. It is also important to understand who verifies the submissions, who uses the data and their views on the program. Of the surveyed users, most participate to contribute to scientific data collection (88%), because of a worry about bees and a desire to help save them (80%), to learn more about species in their property (63%) or region (56%), and because of a personal interest (59%). About 77% report increased awareness of species diversity, while 84% report improvement in their identification skills. We found that 81% had at least one college or university degree. There were more respondents from suburban and rural areas than urban areas, but area did not affect numbers of submissions. While half were between 45 and 64 years of age, age did not influence motivation or number of submissions. Respondents were happy with the program, particularly the website resources, the contribution to knowledge and conservation efforts, the educational values, and the ability to get identifications. Areas for improvement included app and website functionality, faster and more detailed feedback, localized resources, and more communication. Most respondents participate rarely and have submitted fewer than ten records, although about five percent are super users who participate often and submit more than fifty records. Suggested improvements to the program may increase this participation rate. Indeed, increased recruitment and retention of users in general is important, and advertising should promote the outcomes of participation. Fifteen experts responded to a separate survey and were favorable of the program although there were suggestions on how to improve the verification process and the quality of the submitted data. Suggested research questions that could be asked or answered from the data included filling knowledge gaps (species diversity, ranges, habitat, phenology, floral associations, etc.), supporting species status assessments, effecting policy and legislation, encouraging habitat restoration and management efforts, and guiding further research. However, only about half have used data from the project to date. Further promotion of Bumble Bee Watch and community science programs in general should occur amongst academia, conservationists, policy makers, and the general public. This would help to increase the number and scope of submissions, knowledge of these species, interest in conserving them, and the overall program impact.

## Introduction

Community science, which is also commonly known as citizen science, is a fast-growing field where participants commonly collect and/or analyse data as part of a scientific study, often overseen by professional scientists (Silvertown, 2009; Griffin Burns & Harasimowicz, 2012; Miller-Rushing, Primack & Bonney, 2012; Birkin & Goulson, 2015). These projects can deliver baseline or monitoring data, answer research questions, provide skills training and specific knowledge, increase stewardship and awareness, and influence conservation actions and policies (Bonney et al., 2009b; Wiggins & Crowston, 2011; Conrad & Hilchey, 2011; Griffin Burns & Harasimowicz, 2012; Newman et al., 2012; Danielson et al., 2014; Follett & Strezov, 2015; Jordan et al., 2016; Thomas, 2016; Le Féon et al., 2016; Acorn, 2017). While the majority of community science projects involve a biological topic, other examples exist ranging from other sciences (e.g., astronomy, atmospheric sciences) to traditional ecological knowledge, model building, monitoring traffic, medical fields, and informational sciences (Wiggins & Crowston, 2011; Shirk et al., 2012; Follett & Strezov, 2015). Yet the need to create community science frameworks that are successful and sustainable is consistent across all types of projects.

An important part of any successful community science project is an understanding of who the participants are, their reasons for participating, and how their experiences have been to date (Bonney et al., 2009a; Bonney et al., 2009b; Silvertown, 2009; Berg, Dann & Dirkx, 2009). Community science volunteers, particularly those in nature-focused programs, participate for many reasons, such as to learn more about the world around them (Bonter et al., 2012; Griffin Burns & Harasimowicz, 2012; Shirk et al., 2012; Trautmann et al., 2012; Russell, 2014; Van der Wal et al., 2016), to contribute to our overall body of knowledge (Dickinson & Bonney, 2012; Griffin Burns & Harasimowicz, 2012; Shirk et al., 2012; Trautmann et al., 2012; Russell, 2014), to aid in conservation efforts (Shirk et al., 2012; Van der Wal et al., 2016), to increase personal skills and abilities (Bell et al., 2008; Bonter et al., 2012; Dickinson & Bonney, 2012; Shirk et al., 2012; Van der Wal et al., 2016), and/or to simply spend time in nature or with like-minded people (Bell et al., 2008). Individuals who have participated in nature-related projects often participate in other nature-related activities (Federal Provincial and Territorial Governments of Canada, 2014), and many community scientists have knowledge or skills in the area they volunteer (Bell et al., 2008; Bonter et al., 2012; Cooper, Hochachka & Dhondt, 2012; Hames, Lowe & Rosenberg, 2012; Miller-Rushing, Primack & Bonney, 2012; Shirk et al., 2012). While anyone can be a community scientist, participants tend to be highly educated, white, female, and late middle aged through the early senior years (Bonter et al., 2012; Purcell, Garibay & Dickinson, 2012; Toomey & Domroese, 2013). They tend to participate infrequently, submitting few records per person (Worthington et al., 2012; Silvertown et al., 2013; Silvertown et al., 2015; Kelling et al., 2015; Domroese & Johnson, 2017).

There are often spatial biases in community science programs with more data coming from heavily human populated areas than less populated areas (Cooper, Hochachka & Dhondt, 2012; Van der Wal et al., 2016) as there are more potential participants there and it may be easier for them to participate close to their homes (Davies et al., 2011; Van der Wal et al., 2016, although there are exceptions (Cooper, Hochachka & Dhondt, 2012). As well, while individuals may have strong conservation views regardless of where they live (Lutz, Simpson-Housley & De Man, 1999), some studies have found that urban dwellers may have stronger environmental and conservation views (Berenguer, Corraliza & Martin, 2005; Olive, 2014; Olive, 2015). Different regions and audiences also may need different approaches to outreach and education (Bubela et al., 2009; Nisbet & Scheufele, 2009; Chu, Leonard & Stevenson, 2012).

Bumble Bee Watch is a community science program run across Canada and the United States in collaboration by the Xerces Society for Invertebrate Conservation, York University, Wildlife Preservation Canada, and other organizations. It collects information on (Hymenoptera: Apidae: *Bombus*) species phenology, distribution, and more, with the goal to use these data to help conserve at-risk bumblebees. It was launched in 2014 with a web-based platform (http://www.bumblebeewatch.org), with mobile applications for iOS and Android launched in 2017 and 2018 respectively. Based on the idea that bumble bees may, in many cases, be identifiable to species from photographs (Lye et al., 2011; Suzuki-Ohno et al., 2017; Falk et al., 2019), participants take photos of bumble bees and submit through the web or app, along with a date observed and location information. They have the option to use an interactive key or smart filter to assign species name (pre-limited by location), and regional experts verify the identifications. The website also includes resources for the general public and conservationists, including an increasing photo gallery of verified species images, and allows individuals to view data for species, months, areas, and/or to request data for specific project needs.

We wanted to compare metrics on the Bumble Bee Watch program participants, including their knowledge about bumble bees, experience with bumble bee identification, and general feedback, in order to compare it to other community science programs. While Bumble Bee Watch data is currently being used by researchers, such as for species conservation assessments (Szymanski et al., 2016; Committee on the Status of Endangered Wildlife in Canada, 2018; MacPhail, Richardson & Colla, 2019) and regional checklists (Gibbs et al., 2017), we also wanted to know how experts in the field of bumble bee biology and conservation perceive the program, including their experiences with verifying data for the program and actual or potential uses of the data. With over a quarter of our North American bumble bees in decline (IUCN, 2019), and continuing stressors identified (Cameron & Sadd, 2020; Soroye, Newbold & Kerr, 2020), this program has the potential to help contribute data for conservation efforts. However, not all community science programs are successful, failing or being shut down due to a variety of reasons, from poor study design to a lack of funding (Conrad & Hilchey, 2011; Dickinson & Bonney, 2012; Silvertown et al., 2013; Hannibal, 2016; Acorn, 2017). One very important area that can be neglected is the need to ensure that volunteers have a good experience and have their expectations for participating met (Silvertown, 2009; Conrad & Hilchey, 2011; Chu, Leonard & Stevenson, 2012; Fitzpatrick, 2012; Greenwood, 2012; Purcell, Garibay & Dickinson, 2012; Silvertown et al., 2013).

Our main research questions were:

1. What motivates Bumble Bee Watch participants to participate in the program?
2. Are the demographics of participants and participation rates similar to other community science programs?
3. Are there more participants and submissions from urban areas than rural or suburban ones?
4. Do individuals from urban areas have stronger views related to conservation than suburban or rural ones?
5. Do participants increase their knowledge about bumble bees after participating in the program?
6. Do participants increase their skill in bumble bee identification after participating in the program?
7. Have experts used Bumble Bee Watch data in their own research to date?
8. What aspects of the Bumble Bee Watch program are working well, and which ones can be improved?

## Methods

### Design of user survey

The user survey consisted of demographic questions and questions about users’ motivations to participate in and feedback about Bumble Bee Watch. There were 21 questions in total (see Article S1 for the entire survey), based on those discussed in the evaluation of other community science programs, such as The Great Pollinator Project (Domroese & Johnson, 2017), eBird program (Wood et al., 2011), and mammal monitoring study (Newman, Buesching & Macdonald, 2003), amongst others (e.g., reviews in Silvertown et al., 2013; Lewandowski & Specht, 2015). Response options, such as motivations for participation, were generated based on these evaluations as well as informal discussions with and feedback from Bumble Bee Watch participants at training workshops and via e-mail and social media communications. As there is no consistent definition for urban, suburban, and rural areas (see discussion in du Plessis et al., 2002; Ratcliffe et al., 2016), respondents were asked to self-identify based on their perceptions of their communities. The survey was administered using Google Forms from January 23 to February 28, 2018.

Participants were recruited to complete the voluntary user survey through the Bumble Bee Watch e-newsletter and links to the newsletter and survey posted on social media. Respondents were not required to have previously submitted observations to Bumble Bee Watch but previous participation experience was implied. A total of 342 individuals responded to the survey and gave consent for their participation and use of data by reviewing a consent letter and checking a consent box (see Article S1). The number of respondents was a small percentage of the total number of registered and active Bumble Bee Watch users (2.1% of registered Bumble Bee Watch users (342/16,055) and 5.4% (342/6,292) of all users having submitted at least one record).

### Design of expert survey

The expert survey consisted of fewer demographic questions, and more questions related to community science research and its application, as compared to the user survey (see Articles S2 and S3 for survey and consent form).

The expert survey participants were determined by the authors. The initial list of expert participants was based on Bumble Bee Watch verifiers but was revised to reflect those most applicable or best suited to respond, including others in the field. Invitations to complete the Google Forms expert survey were sent out by e-mail in February and March 2018 to these 15 individuals. To increase sample size, an additional 17 experts were approached in October 2019. The survey these experts received (see Article S3) was identical to the one sent out in 2018 (see Article S2) with the exception of the removal of the invitation and link to complete the general user survey. In total, 32 experts were invited to participate, and 15 responses were collected.

This survey of users and experts was approved by York University’s Faculty of Environmental Studies (November 2017, no reference number (internal approval)) and the data re-approved for use by York University’s Office of Research Ethics (September 2019, ref# STU 2019-097).

## Data from Bumble Bee Watch Program

An export of the main Bumble Bee Watch program database, including both pending, tentative, and verified nest and bee records, was obtained on February 28, 2018. Summaries were calculated in Excel (Microsoft Office 365 ProPlus, Version 1909) to compare responses obtained in the user survey to metrics from the database, such as the relative number of contributors and the number of submissions per person and per province/state/territory (“jurisdiction”). The number of registered users of Bumble Bee Watch was provided by Rich Hatfield of the Xerces Society for Invertebrate Conservation to VJM on February 28, 2018; this number differs from that of the number of contributors to the Bumble Bee Watch database as not everyone who had created an account had submitted records.

### Comparison demographics from Canada and the United States

To allow for comparisons of Bumble Bee Watch respondents to the broader public, demographic data was obtained for both Canada and the United States through their respective census agencies. This included population by age group, federal jurisdiction, and education (Statistics Canada, 2016; Statistics Canada, 2017a; United States Census Bureau, 2017a; United States Census Bureau, 2017b).

### Coding, analysis of data

Data collected through the surveys were exported from Google Forms into an Excel spreadsheet. Blank values or skipped responses were removed for the analyses of that question. Basic summaries were completed in Excel, while statistical analyses were carried out using SPSS ver 24 (IBM Corp 2016). Kruskal-Wallis tests were used to investigate if the number of submissions or motivations varied depending on the number of years the respondents have been involved in the program, the age of respondents, or the area (urban, suburban, rural) they lived in. To control for false discovery rates we used the Benjamini–Hochberg procedure to adjust the *p*-values (Pike, 2011) for all analyses involving the same variable (i.e., years involved, age, area). Dunn’s post-hoc pair-wise comparisons were run when significant differences ( *p* < 0.05) were found, with the resulting significance values adjusted by the Benjamini–Hochberg procedure. Note that the variables tested (e.g., number of submissions, years involved, age) were collected as factors or categories (e.g., <10 submissions, 10-20 submissions, etc.) and not on a numerical range.

Questions 10 (bumble bee training), 11 (wildlife identification skills) and 16 (education) on the user survey and Questions 9 (used data from program) on the expert survey had an open-form “other” response option. These responses were re-classed into the provided category options for each question as applicable (i.e., the open-form text responses were coded into one or more of the originally provided response options). For example, for Question 10, responses such as “I have been taking pictures of bumble bees for years” and “I’m a beekeeper (*Apis mellifera*)” were classified as “no” for specialized training in bumble bees, while responses such as “We did a year long project on native bumble bees in our area and spent a year learning to identify them, including meeting with experts, such as T’ai Roulston, UVA, and Sam Droege” and “Field Experience with [Wildlife Preservation Canada]” were classified as yes.

Qualitative open-form responses were coded and then categorized (Strauss & Corbin, 1998; Saldana, 2009; Newing, 2010). The number of individuals who made statements captured by each first level descriptive code and second level axial category were summed, which permitted quantitative analyses.

Analyses, results and discussions on the surveys related to the perceived ease of species identification for both users (Questions 19, 20) and experts (Questions 14, 15) is discussed in MacPhail et al. (unpublished data).

## Results

### User survey

A total of 342 individuals responded to our user survey. An overwhelming majority of respondents participate in Bumble Bee Watch to contribute to scientific data collection (88.3%) and because they were worried about bees and want to help save them (80.3%) (Table 1). This is followed by a majority wanting to learn more about species on their property or region (62.6% and 56.1% respectively), and because they have a personal interest in bumble bees (59.4%), among other motivations (Table 1). For those who wrote in other motivations (25 individuals), 36% indicated they participate because they want to share information with others, 16% participate to improve mental health, be part of natural world, or enjoy a hobby, and 12% participate because they wanted to attract and support pollinators, particularly with their own gardens. Note that individuals could have multiple motivations.

**Table 1:** Motivations for participating, as selected by user survey respondents from a provided list of options. Respondents (*n* = 342) could select more than one motivation so numbers do not add up to 342 respondents or 100%.

Motivations	Number of respondents	Percent of respondents
To contribute to scientific data collection	302	88.30
I’m worried about bees and want to help save them	284	83.04
I want to learn what species are on my property	214	62.57
I have a personal interest in bumble bees	203	59.36
I want to learn how to identify the biodiversity in my region	192	56.14
To share the uncommon or rare species I find	126	36.84
Recreational learning/Family activity	120	35.09
Participation in special events (e.g., Great Canadian Bumble Bee Count or a Bioblitz)	47	13.74
The preservation of ecological diversity	1	0.29

There were no significant differences in any motivations for participation as compared to age of respondents (various K-W tests, *df* = 7, *p* > 0.05; Table S1), years of participation (various K-W tests, *df* = 4, *p* > 0.05; Table S1), or area lived in (various K-W tests, *df* = 2, *p* > 0.05; Table S1).

More than three quarters (77.2%) of respondents report an increased awareness of bumble bee species diversity, and 84.8% report probable improvement in their identification skills as a result of participation in Bumble Bee Watch (63.5% yes, 21.3% maybe, 7.6% no, and 7.6% I don’t know). Most had only moderate to low confidence in their identification skills (2.9% said very confident, 12.0% said confident, 41.8% said somewhat confident, 40.9% said not confident, and 2.3% were unsure). This may be explained by the amount of training they have had with bumble bee identification, with the majority (89.5%) reporting no specialized training; 7.9% had some training, 2.0% may or may not have had training, and 0.6% did not answer the question. However, over 85% of respondents indicated they had some (62.3%) or many (22.8%) other wildlife identification skills, while 0.9% said just identification skills with bumble bees, and 14.0% said they had no skills at all (0.6% did not respond).

Of the 87 respondents who had specified specific wildlife identification skills in the optional text box, the majority had experience with birds (60.9%), followed by plants (39.1%), lepidopterans (26.4%), other insects/invertebrates (18.4%) (excluding other bees and odonates that were each reported 11.5%), general species identification (14.9%), mammals (13.8%), amphibians (4.6%), reptiles (3.4%), fish (2.3%), and three other groups (1.1% each) (numbers can add to >100% as multiple responses could have been recorded per person).

More than half of the survey respondents have been involved in naturalist, gardening, or wildlife groups, being currently active (51.2%) or active in the past (8.8%), while 39.5% have not been members, and 0.6% did not respond.

Many of the respondents (*n* = 210) gave feedback about ways to improve Bumble Bee Watch, with the most common category being IT related (41.9% of respondents), followed by improved feedback (27.6%), and additional tools and resources (19.1%), although 15.7% indicated that they were happy and had no further suggestions (Table  S2).

Two hundred and fifty-five respondents provided feedback on what they liked best about the program. The top four best liked features included the resources on the website (38.8%), the contribution to their own and more general knowledge and conservation efforts (including policy) (33.7%), the educational values (24.7%), getting identifications and feedback on submitted records (18.0%) (Table 2).

**Table 2:** Categories of feedback about what respondents (*n* = 255) to the user survey liked best about the Bumble Bee Watch program. Note that respondents could make multiple suggestions so the total of all categories do not add up to 100%. Free-form responses were initially coded into one of 31 categories by three individuals, and then further collapsed into these seven categories. Respondents who provided feedback that was not related to the question were excluded.

**Program features liked best**	**Number of respondents**	**Percent of respondents**
Resources on the website (including identification keys, data/records, photos, maps)	99	38.82
Contributing to both user and general knowledge, and contribution to conservation efforts about bumble bees, incl helping to influence policy	86	33.73
Educational - helps with learning about bumble bees	63	24.71
Identification and feedback on submitted records	46	18.04
Fun, family-friendly outdoor based hobby, community of users	45	17.65
Easy to use, works great	33	12.94
Availability of app	7	2.75

Many respondents were from suburban areas (39.5%), followed by rural (34.2%) and finally urban (26.3%) areas. Most respondents had higher education with 81.0% having at least one post-secondary degree (college through PhD), while a further 12.9% had some college or university, 1.8% vocation/trade training, 3.5% high school diploma, and 0.6% some high school.

Each of the age classes from age 12 to 75+ were represented in our survey. Of the respondents, 50.9% were in the 55–64 and 45–54 age groups, which is more than twice the relative percent of the US and Canadian population in those two categories (Table 3). Only 1.75% of respondents were between 12 and 24 years of age, which is less than a quarter of the relative corresponding national populations (Table 3).

**Table 3:** A comparison of the Bumble Bee Watch user survey respondents’ age groups to the combined United States and Canadian populations in 2016 (Statistics Canada, 2016; United States Census Bureau, 2017a). Note that the age groupings varied between the survey and the national demographics for the two youngest classes. Total number of respondents to the survey was 342. The total population aged 10-75+ was 280,705,770 in the United States and 17,858,089 in Canada, for a combined total of 298,563,859.

Age group	Percent of user survey respondents	Percent of US and Canada combined population
12 to 17 (survey) or 10 to 19 (US & Canada)	0.29	15.55
18 to 24 years survey or 20 to 24 year (US & Canada)	1.75	8.20
25 to 34 years	8.19	15.75
35 to 44 years	15.20	14.48
45 to 54 years	21.05	15.21
55 to 64 years	30.99	14.23
65 to 74 years	19.88	9.58
75 plus	2.63	7.00

Respondents were closely split between geographic regions: 197 (57.6%) from the Eastern Region and 145 (42.4%) from the Western Region. The relative percent of respondents per province/state/territory (“jurisdiction”) is similar to the relative percent of records per region that have been submitted to the database with only three jurisdictions having >2% difference in relative percent between the respondents and the database (Colorado, Ontario, Washington) (Table S3). However, 14 (28.6%) jurisdictions had >2% difference in relative percent between respondents and the national population (Table S3) (Statistics Canada, 2017b; United States Census Bureau, 2017a). A total of 49 jurisdictions were represented by respondents, while records exist in Bumble Bee Watch for these and 13 others (Table S3). A third of respondents (33.0%) had been participating with Bumble Bee Watch for two years, followed by those with less than one year of participation (23.7%), one year (20.5%), three years (16.5%), or all four years since the program launched in 2014 (6.4%). However, only a few respondents submit records often (7.0%); most reported only rarely (43.0%) or sometimes (42.7%) submitting photos, with a few submitting only if it is a rare or uncommon species (7.3%). Indeed, more than half of respondents (69.0%) have submitted less than 10 records, although this is lower than the percent of Bumble Bee Watch contributors who also submitted less than 10 records (94.2%) (Table 4).

**Table 4:** The relative percent of Bumble Bee Watch user survey respondents (342 individuals) and program contributors (6,292 unique individuals across both nest and bee records, as of February 28, 2018) per number of records submitted to the Bumble Bee Watch database.

Number of records submitted to Bumble Bee Watch	Percent of user survey Respondents	Percent of database contributors
Less than 10	69.0	94.2
10–20	17.0	3.8
More than 20	8.8	1.3
More than 50	5.0	0.8
(blank)	0.3	n/a
Grand Total	100.0	100.0

There is a significant difference in the number of records (as divided into groupings) made to Bumble Bee Watch as compared to the years of involvement (K-W H statistic = 55.313, *df* = 4, *p* < 0.001), with a greater number of records being submitted per accumulated years of participation, except between those who had zero and one or zero and two years of experience (see Table S4). There was no significant difference in the number of photos submitted by age group (K-W H statistic = 5.983, *df* = 7, *p* = 0.774) or by type of area (urban/suburban/rural) (K-W H statistic = 1.009, *df* = 2, *p* = 0.755).

### Expert survey

Fifteen individuals responded to the expert survey: eight from the Eastern region (east of the Mississippi in the United States, and east of Ontario (including Ontario) in Canada) and seven from the Western region (west of the Mississippi in the United States and west of Manitoba in Canada). At the time of their responses (April 2018 or November 2019), 60% of the respondents had verified records on Bumble Bee Watch directly or indirectly, with 80% of the remaining five experts willing to verify in the future. Almost 3/4 of the expert respondents (73.3%) had not submitted records to Bumble Bee Watch, but about half (46.7%) have participated in other community science programs.

About half (46.7%) of the experts have used Bumble Bee Watch data in their research. Example usage included “[collecting] information from Bumble Bee Watch for Special Concern Species in WI [Wisconsin] for the WI DNR [Department of Natural Resources]. This information gets used for conservation of the species”, “looking up records for *B. affinis*”, “[using] recent sightings…for site selection”, and for published manuscripts on species ranges and conservation status. All expert respondents provided examples of research questions that Bumble Bee Watch data could help to answer, including those related to species ranges, phenology, floral associations, detection and monitoring, and more (Table 5).

**Table 5:** Ideas of questions that Bumble Bee Watch data can be used to answer, according to respondents to the expert survey (*n* = 15). Free-form responses were summarized and coded to get to these categories.

Categories	Number of respondents	Percent of respondents
Species ranges, including current distributions, range contractions and expansions, species distribution models	13	86.67
Phenology for each bee species, including different castes	7	46.67
Collecting information on rare species, comparing ratios and trends in ratios of common to uncommon species, investigating changes in diversity, and calculating species status/extinction risk	5	33.33
Floral associations, including changes in	4	26.67
Understanding species diversity and habitat preferences	2	13.33
Monitoring, detection and prevalence studies	2	13.33
“So many” [ways data can be used]	2	13.33
To see if participation in Bumble Bee Watch affects participant behaviour	1	6.67
To feed into studies looking at different factors to see if they affect bumble bees (e.g., climate change, habitat loss)	1	6.67
Machine learning based identifications	1	6.67
Bee identification success rates	1	6.67
Identification of sites for research and restoration work	1	6.67

Ten of the expert respondents provided open feedback on ways to improve submitting, verifying and/or using records, which was then condensed down into four categories. The most frequent comments related to app or website features (e.g., being able to take a photo from main screen of app rather than from a species page, uploading multiple images at once, making the interface faster, downloading the data easier). This was followed by ways to improve the original data submission (e.g., instruct users to submit photos showing key identification features, autopopulate the location field based on metadata in photo, capture additional habitat information) and verification process.

## Discussion

Bumble Bee Watch participants are highly motivated by the desire to contribute to scientific data collection and to help save bumble bees, as well as to further their own education, such as learning about local species, and for social reasons, such as an activity to participate in with family and friends and/or as part of a special event, in addition to general enjoyment. These motivations were consistent across ages of participants, years of participation, and areas they live in. Most of the participants reported becoming more aware about bumble bee diversity and improving their identification skills after participating, and the top feature of the program they liked were the resources on the website. To keep the program current and sustainable, it is important to understand and continue to motivate participants.

These are all common motivations in community science projects (Van Den Berg, Dann & Dirkx, 2009; Bonney et al., 2009a; Bonney et al., 2009b; Bonney & Dickinson, 2012; Chu, Leonard & Stevenson, 2012; Newman et al., 2012; Shirk et al., 2012; Silvertown et al., 2013). Our results also show that the desire for bumble bee conservation is as high as the general contribution of scientific data; participants could be given more concrete ways that they can help and/or they could be recruited by other projects to help move conservation efforts forward in their areas. Bees are popular animals right now, and The Great Pollinator Project (Domroese & Johnson, 2017), found that volunteers ranked the desire for education and learning about pollinators to be the number one motivation followed by the participation in a community science project.

These results are consistent with other research which show programs with a nature-based theme often recruit individuals who care about the environment and wish to take action to help, unlike broader public education programs where participants are motivated by other reasons, such as career advancement (Guiney & Oberhauser, 2009; Van Den Berg, Dann & Dirkx, 2009; Newman et al., 2012; Lewandowski & Oberhauser, 2017). Regardless, it is important for each project to recognize the motivations driving their volunteers and to ensure these desires are met in order to keep interest and thus retention high (Bonney et al., 2009b; Bonney et al., 2009a; Silvertown, 2009; Conrad & Hilchey, 2011; Chu, Leonard & Stevenson, 2012; Fitzpatrick, 2012; Greenwood, 2012; Purcell, Garibay & Dickinson, 2012; Silvertown et al., 2013; Domroese & Johnson, 2017). In the case of Bumble Bee Watch, this can be accomplished by showing the participants the scientific value of their data (e.g., frequent summaries of the data and examples of how it has been used, such as through selected publications) and how it is helping to meet the project goals (e.g., where it has been used in species at risk policy designations, conservation decisions), in addition to providing more educational resources to the participants (e.g., tips on creating nesting habitat, best flower species to plant, guidance on how to reach out to decision makers). This has already been occurring through the use of an e-newsletter and blog hosted by The Xerces Society for Invertebrate Conservation (2020) but more frequent and regional communications would be beneficial to keep interest high.

It is also important to improve the user experience with the program to encourage continued participation, from the initial recruitment and registration through reporting and retention stages. Community science projects traditionally have low participation versus registration rates, and low contributions on a per person basis (Worthington et al., 2012; Silvertown et al., 2013; Silvertown et al., 2015; Kelling et al., 2015; Domroese & Johnson, 2017); Bumble Bee Watch is no exception. About 39% of registered Bumble Bee Watch users submitted at least one record, which is higher than other programs, such the 25% of registrants in the Great Pollinator Project (Domroese & Johnson, 2017) and 30% of those who contacted organizers in the Protea Atlas Project (Silvertown et al., 2013).

Virtually all (94%) of the Bumble Bee Watch database contributors contributed only a few (<10) records, with only a few (0.3%) of participants submitting >100 records (17.1% of the data). This was also seen among our survey respondents. Similarly, the Protea Atlas Project found that only 20% of their participants submitted more than 50 site records, and 2.5% more than 1,000 (Silvertown et al., 2013); with the eBird program, 10% of the participants submitted 90% of the records (Wood et al., 2011). These are low numbers of active users but quantity of participants and quality of the resulting dataset are not always equal. These super volunteers or power users often contribute high amounts of high quality data to a program that can help to offset the low participation; these individuals should be especially targeted for retention (Wood et al., 2011; Hames, Lowe & Rosenberg, 2012; Silvertown et al., 2013; Kelling et al., 2015; Hannibal, 2016).

Programs generally want to increase the number of new participants each year to both grow the program and offset participants who do not return. For example, FeederWatch has a 70% annual retention rate, but needs 3,000 to 4,000 new participants annually to keep participant numbers and geographic coverage consistent (Bonter et al., 2012). It could be expected that distribution of years of participation would be greatest numbers in the most recent cohort and lower numbers each year. This was seen in the Great Pollinator Project where 41%, 36%, 17%, and 7% of survey respondents participated in one to four years, respectively, of the project (Domroese & Johnson, 2017), but the greatest number of our Bumble Bee Watch survey respondents were not from under one year or first year participants but for second years (33%), although the fewest numbers of respondents (6.4%) had been the early adopters, participating for four years. This may be a feature of program roll-out and advertisement being stronger at the time these second-year participants started; further investigations into the actual numbers of new and returning users as compared to promotion efforts would be needed to better identify any correlation. However, it is clear that volunteer retention is key; indeed, Sheppard et al. (2017) believe retention is a measurement that can account for both data quality and volunteer engagement.

Our user survey respondents had a number of similar responses in terms of what they liked about the program and what could be improved. Commonly stated areas for improvement related to their motivation to learn and to see that they are helping science. Users wanted to get faster feedback on their submissions, more tools and resources to identify and attract pollinators, and increased communication to learn what others have been finding and how the data has been used. In regards to the communications with others, in-person workshops and direct contact with experts are not always possible, but there may be a role for virtual training and online community discussion forums in sharing information and experiences and getting feedback and advice (Bonney & Dickinson, 2012; Bonter et al., 2012; Triezenberg et al., 2012; Toomey & Domroese, 2013; Austen et al., 2018).

The Great Pollinator Project also struggled with meeting the desired speed of feedback and sharing of data to users, particularly for higher level summaries and reports that can take a long time before analyses can be completed, and encouraged program leads to find solutions for this common problem (Domroese & Johnson, 2017). While providing feedback can be time intensive, the Bee Watch program developed an automated natural language generated feedback program that immediately gave generalized feedback and information to users after expert review based on the species being (mis)identified (Blake et al., 2012; Van der Wal et al., 2016). The Bee Watch program found that this automated feedback greatly increased participant learning, skill, and retention (Blake et al., 2012; Van der Wal et al., 2016). The Evolution MegaLab project gave immediate automated feedback to users upon record submission, including text and figures comparing their submission to historic records in the same area, which also helped to motivate their participants (Worthington et al., 2012). Several community science programs, such as iNaturalist, Pl@ntNet, and Merlin Bird ID, use an artificial intelligence and deep learning computer programs to suggest identifications based on comparison to other photos in their database and nearby records , which provides instant feedback to users (Wäldchen & Mäder, 2018; Ceccaroni et al., 2019). The use of new technologies, including new software programs, will help to remedy some currently existing challenges in both Bumble Bee Watch and community science programs in general to improve data collection and the user experience going forward (Newman et al., 2012; Wäldchen & Mäder, 2018; Ceccaroni et al., 2019).

The areas of improvement noted by Bumble Bee Watch users were also commonly the main areas users enjoy with the program, such as the resources available on the website and their ability to help save the bees through the sharing of their data. This suggests that although more can be done, such as customized regional bee identification workshops or pollinator-friendly gardening guides, program organizers are on the right track with the provided features. We do not discuss the accuracy or perception of user and expert identification in this paper as has been done for other bumble bee community science programs (Lye et al., 2011; Suzuki-Ohno et al., 2017; Falk et al., 2019), but this information (including commonly mis-identified species) would also help to improve the program and data quality (but see MacPhail et al. (unpublished data) for this).

Our survey was conducted in the winter months when bumble bees are not active across most of North America, and submissions to the program are thus low. It is possible that users might have had slightly different opinions, or different rankings of opinions, than if we had done the survey during the peak of the active season. For instance, respondents may have forgotten difficulties such as potential issues with the submission process but been more aware of the delays in getting verification of their photos. However, as some respondents had multiple years of participation to reflect on and some submit their photos throughout the fall and winter, and as a wide variety of responses were recorded across the participants, any temporal-related biases in our results are likely low.

The experts who responded to our survey were generally pleased with the Bumble Bee Watch program, although they also had suggestions on how to improve the program interface and quality of the data. The main areas that they believed the data could be used for included filling knowledge gaps, supporting provincial and federal assessments, effecting policy and legislation, encouraging habitat restoration and management efforts, and guiding further research. However, despite the many areas of potential use, only about half of the experts have actually used Bumble Bee Watch data in their research. The advertisement of the dataset availability and example questions may be worthwhile for Bumble Bee Watch to share with researchers and conservationists through formal academic means and social media. As the program and dataset grow, more avenues to use the data will also evolve. Indeed, Bumble Bee Watch data is routinely accepted into the Bumble Bees of North America database managed by Dr. Leif Richardson and the USGS BISON (Biodiversity Information Serving Our Nation) database, and has also been added to other national and regional databases, so the data is increasingly becoming available to researchers.

Over half of our user survey respondents were middle-aged, between 45 and 64 years of age, which is common of many programs as they include the group who often have more time to volunteer as they have older children and more stable employment or have retired (Guiney & Oberhauser, 2009; Purcell, Garibay & Dickinson, 2012; Toomey & Domroese, 2013; Domroese & Johnson, 2017; Sheppard et al., 2017). Interestingly, we had more individuals that were 75 years of age and older respond to our survey than those under 24 years of age. While we had few respondents in the latter group, this may be because most youth under 18 would only participate with an adult and/or under an adult’s account. Yet there are benefits for youth participation, not only to get them started on a life-long love of nature and contribution to community science and conservation projects but also because they can often be single-focused and energetic, and be more skilled than adults in some areas (Wells & Lekies, 2006; Guiney & Oberhauser, 2009; Griffin Burns & Harasimowicz, 2012; Silvertown et al., 2013). Our results show that there was no difference in the number of records submitted per age group. Our program encourages people to get outside and be active while enjoying their local gardens or trails, which is a necessary part of having a healthy physical, mental, and social life, especially for older adults who tend to become inactive over time (Buchner et al., 1992; Wagner et al., 1992; Phillips, Schneider & Mercer, 2004; Bushway et al., 2011).

While every province, state and territory that have bumble bees were represented in the Bumble Bee Watch database, some of these regions have low participation rates as compared to the relative proportion of the population and to the entire program as a whole. Increased promotion of the program in these areas, including through targeted presentations, ”bee walks”, and dedicated survey programs could help to increase data coverage. For example, one of us (VJM), gave a public presentation, bee walk, and CBC media interview on Prince Edward Island in August 2019, and in the following three months the number of submissions had almost doubled (1.8 times) what had been submitted from the province in the previous five and a half years combined; in fact, 25.5 times more records were submitted in the three-month August through October 2019 period as compared to the average of the same three months in the preceding five years. After a single CBC media interview in New Brunswick in August 2019, about 5.8 times the number of records were submitted in August through October time period compared to the average of those months over the previous 5 years. General advertising should account for the motivations of residents from different types of areas of physical and demographic communities, such as advertising the ability to find and share rare species to those in rural and suburban areas or to participate in special events to those in urban areas. Although there was no difference in the number of records submitted based on the area in which people live, urban residents had the least number of respondents in our survey; it is possible that this is due to the erroneous belief that cities do not contain bumble bees or that they are already well surveyed: advertising could help alleviate these concerns. As we did not provide definitions as to what constituted urban, suburban, or rural, it is also possible that some respondents identified with the suburban category instead of the urban category, explaining the lower number in the latter group.

While there are challenges with community science programs, there is an increased understanding of the need for good practices, from data collection and validation to participant communications and retention (Bell et al., 2008; Silvertown, 2009; Conrad & Hilchey, 2011; Cooper, Hochachka & Dhondt, 2012; Phillips, Bonney & Shirk, 2012; Worthington et al., 2012). The development of appropriate protocols and supports (administrative, technological, and financial), can overcome the challenges and ensure the positive, long-lasting outcomes of these programs (Conrad & Hilchey, 2011; Cooper, Hochachka & Dhondt, 2012; Dickinson & Bonney, 2012; Pocock et al., 2015).

## Conclusion

Bumble Bee Watch has the potential to engage the public, answer research questions, and increase the conservation of bumble bees across the United States and Canada through avenues resulting from having thousands of participants of various ages from rural through urban locations increasing their understanding of bumble bee diversity and their desire to conserve them while generating the data for research and conservation purposes via photo submissions of the bumble bees they see across the landscape. Improvements are needed in order to maximize volunteer retention and data quality, which could include the adoption of new technology such as automated feedback and the provision of more targeted resources such as identification guides and species lists. Further promotion of community science programs amongst academia, conservationists, policy makers, politicians, and the general public should occur in order to increase the number of participants and submissions (both geographically and temporally), the use of the data amongst a broader group of stakeholders and finally, our knowledge of species and our interest in conserving them.

##  Supplemental Information

10.7717/peerj.9141/supp-1 Data S1 Original responses from user survey

10.7717/peerj.9141/supp-2 Data S2 Original responses from expert survey

10.7717/peerj.9141/supp-3 Data S3 Excerpt of Bumble Bee Watch database used in comparisons to survey responses

10.7717/peerj.9141/supp-4 Article S1 Copy of User Survey Administered via Google Forms in 2018

10.7717/peerj.9141/supp-5 Article S2 Copy of Expert Survey Administered via Google Forms in 2018

10.7717/peerj.9141/supp-6 Article S3 Copy of Expert Survey Administered via Google Forms in 2019

10.7717/peerj.9141/supp-7 Table S1 Results for a series of Kruskal-Wallis test statistics for the comparisons of user survey respondents’ motivations by age of respondent, number of years participating in Bumble Bee Watch, or by area they live (urban/suburban/rural) Significance levels were adjusted to control False Discovery Rates (FDRs) using the Benjamini-Hochberg procedure.

10.7717/peerj.9141/supp-8 Table S2 Categories where user survey respondents (n=210) suggested improvements could be made to the Bumble Bee Watch program Note that respondents could make multiple suggestions so the total of all categories do not add up to 100%. Free-form responses were initially coded into one of 29 categories by three individuals, and then further collapsed into these six categories.

10.7717/peerj.9141/supp-9 Table S3 A comparison of the relative percentage of user survey respondents, database records, and combined population per United States state or territory and Canadian province or territory (“jurisdiction”) There were 342 survey respondents and 23,017 records in the Bumble Bee Watch database as of February 28, 2018, covering both the United States and Canada. The United States population during the 2015–2017 period was 321,004,407 (excluding Puerto Rico) (United States Census Bureau, 2017a) , and the Canadian population in 2016 was 35,151,728 (Statistics Canada, 2017b) , for a combined population of 356,156,135.

10.7717/peerj.9141/supp-10 Table S4 Statistical output for a series of Dunn’s tests comparing the total number of years the user survey respondents have participated in Bumble Bee Watch to their reported number (class) of submissions Significance levels were adjusted to control False Discovery Rates (FDRs) using the Benjamini-Hochberg procedure.

## Additional Information and Declarations

### Data Availability

The following information was supplied regarding data availability: The anonymized raw survey responses by users and experts, and the anonymized excerpt of the Bumble Bee Watch database which was used as a comparison to survey responses, are available in the Supplementary Files.
