# Supplementary material for: Community science participants gain environmental awareness and contribute high quality data but improvements are needed: insights from Bumble Bee Watch
Source: PeerJ. 2020 May 12;8:e9141. doi: 10.7717/peerj.9141 (PMC7227640; doi:10.7717/peerj.9141)
Supplement: Article S1 [file peerj-08-9141-s004.pdf]

Bumble Bee Watch - User Survey

The purpose of this survey is to help build an understanding of the application of citizen science in biodiversity conservation. As Bumble Bee Watch.org has generated a remarkable database of bumble bee records, researchers would like to know more about the website's users. Learning about participants demographics, motives, and confidence with bumble bee identification is highly useful for researchers who wish to understand the possibly wide-ranging application of citizen science in the future.

* Required

Informed Consent to Participate in Research

Please read the informed consent form and indicate your consent below.

1. **By checking the box below you indicate that you have read the form, below, and consent to participate in the BumbleBeeWatch.org survey conducted by York University research team.** *

   Check all that apply.

   ☐ I consent.

research ethics | YORK UNIVERSITÉ UNIVERSITY
Office of Research Ethics York University Kaneff Tower, Fifth Floor – 4700 Keele Street, Toronto, Ontario, Canada, M3J 1P3 ore@yorku.ca research.info.yorku.ca

Informed Consent Form

Date: September 26, 2017

Study Name: Assessing the Quality of BumbleBeeWatch.org Data

Researcher name:
Faculty of Environmental Studies
York University
Health, Nursing and Environmental Studies Building
4700 Keele St, Toronto, ON
M3J 1P3

Principal Investigator: Dr. Sheila Colla, PhD, srcolla@yorku.ca
Student Researchers: Shelby Gibson, MES candidate, sgibs94@yorku.ca, Madeleine Lavin, MES candidate, mlavin@yorku.ca, Victoria MacPhail, PhD candidate, vmacphail@gmail.com

Purpose of the Research:

You are being asked to participate in a short (5-10 min) survey because you are a user of BumbleBeeWatch.org. York University is a partner on the BumbleBeeWatch program, and are interested in assessing the quality of data collected through citizen science programs, such as BumbleBeeWatch. Part of this research requires an understanding of who is submitting observations to the database, as well as their motives for doing so. We do not foresee any risks or discomfort from your participation in the research. The benefit of participation is that the participant is able to contribute to a greater understanding of the application of citizen science research in biodiversity conservation. There is no requirement to complete the survey, and once a participant has begun the survey they reserve the right to quit at any point. The survey responses remain anonymous unless permission is given otherwise. As this survey is being administered online, we are relying on this informed consent letter (as opposed to a signature form) to fulfill the duty of informed consent. Further, the data collected may be used in a peer-reviewed publication and conference presentations in the future.

Voluntary Participation and Withdrawal:

Your participation in the study is completely voluntary and you may choose to stop participating at any time. Your decision not to volunteer, to stop participating, or to refuse to answer particular questions will not influence the nature of the ongoing relationship you may have with the researchers or study staff, or the nature of your relationship with York University either now, or in the future. In the event you withdraw from the study, all associated data collected will be immediately destroyed wherever possible. Should you wish to withdraw after the study, you will have the option to also withdraw your data up until the analysis is complete.

Confidentiality:

The method of documentation is through an online Google Survey, which will have an

option to export the data collected in to an Excel spreadsheet. The data will be stored on a locked laptop for two years. After this, it may be added to an online data repository. This will allow for the data to be used in future research which may look at citizen science in biodiversity conservation.

Unless you choose otherwise, all information you supply during the research will be held in confidence and unless you specifically indicate your consent, your name will not appear in any report or publication of the research. Your data will be safely stored on a locked laptop, and only the research team will have access to this information. By September 2019, the data will either be destroyed or added to an online data repository. Confidentiality will be provided to the fullest extent possible by law.

The data collected in this research project may be used – in an anonymized form - by members of the research team in subsequent research investigations exploring similar lines of inquiry. Such projects will still undergo ethics review by the HPRC, our institutional REB. Any secondary use of anonymized data by the research team will be treated with the same degree of confidentiality and anonymity as in the original research project. If data is added to a repository, it will be done so in an anonymized manner.

The researcher(s) acknowledge that the host of the online survey (e.g., Qualtrix, Survey Monkey etc.) may automatically collect participant data without their knowledge (i.e., IP addresses.) Although this information may be provided or made accessible to the researchers, it will not be used or saved without participant's consent on the researcher's system. Further, "Because this project employs e-based collection techniques, data may be subject to access by third parties as a result of various security legislation now in place in many countries and thus *the confidentiality and privacy of data cannot be guaranteed during web-based transmission.*

Questions About the Research? If you have questions about the research in general or about your role in the study, please feel free to contact me at sgibs94@yorku.ca , or my supervisor, Dr. Sheila Colla at srcolla@yorku.ca. You may also contact the Graduate Program in Faculty of Environmental Studies at York University, (416)-736-5252.

This research has received ethics review and approval by the Delegated Ethics Review Committee, which is delegated authority to review research ethics protocols by the Human Participants Review Sub-Committee, York University's Ethics Review Board, and conforms to the standards of the Canadian Tri-Council Research Ethics guidelines. If you have any questions about this process, or about your rights as a participant in the study, please contact the Sr. Manager & Policy Advisor for the Office of Research Ethics, 5th Floor, Kaneff Tower, York University (telephone 416-736-5914 or e-mail ore@yorku.ca).

Legal Rights and Signatures:

I__________________________consent to participate in the BumbleBeeWatch.org survey conducted by York University research team. I have understood the nature of this project and wish to participate. I am not waiving any of my legal rights by signing this form. My signature

research ethics | YORK UNIVERSITÉ UNIVERSITY

Office of Research Ethics
York University

Kaneff Tower, Fifth Floor – 4700 Keele Street,
Toronto, Ontario, Canada, M3J 1P3

ore@yorku.ca
research.info.yorku.ca

below indicates my consent.

Signature ______________________ **Date** ______________________
Participant

______________________ November 28, 2017
Principal Investigator

Additional consent:

1. **Consent to waive anonymity**

I, ______________________ consent to the use of my name in the publications arising from this research.

Signature ______________________ **Date** ______________________
Participant Name:

Biogeographic Region

2. **Please select the biogeographic region from which you primarily submit bumble bee observations:** *
Mark only one oval.

- ◯ Eastern Region - United States: East of the Mississippi | Canada: East of Ontario (including Ontario)
- ◯ Western Region - United States: West of the Mississippi | Canada: West of Manitoba (including Manitoba)

Bumble Bee Watch - User Information

These questions are designed to get a sense of how long you have been a user of the site, what your reasons are for participating in this project, how it has helped your bumble bee identification skills, and whether you have any prior experience with wildlife identification.

3. **How many years have you been a user of Bumble Bee Watch.org?** *
Mark only one oval.

- ◯ Under 1 year
- ◯ 1 year
- ◯ 2 years
- ◯ 3 years
- ◯ 4 years

4. **How often do you submit bumble bee observations to Bumble Bee Watch?** *
Mark only one oval.

- ◯ Often
- ◯ Sometimes
- ◯ Rarely
- ◯ Only if it is a rare or uncommon species

5. **How many photos have you submitted to Bumble Bee Watch?**
Mark only one oval.

- ◯ Less than 10
- ◯ 10 - 20
- ◯ More than 20
- ◯ More than 50

6. **Before using Bumble Bee Watch, were you aware of how many bumble bee species there are in your region?** *

Mark only one oval.

- Yes
- No

7. **What is your level of confidence in bumble bee identification?** *

Mark only one oval.

- Very confident
- Confident
- Somewhat confident
- Not confident
- Unsure

8. **Do you think your ability to identify bumble bee species has improved as a result of using Bumble Bee Watch?** *

Mark only one oval.

- Yes
- No
- Maybe
- I don't know

9. **What motivates you to submit your observations to Bumble Bee Watch? Please check all that apply.** *

Check all that apply.

- [ ] To contribute to scientific data collection
- [ ] I want to learn what species are on my property
- [ ] I want to learn how to identify the biodiversity in my region
- [ ] Participation in special events (e.g. Great Canadian Bumble Bee Count; Bioblitz)
- [ ] I have a personal interest in bumble bees
- [ ] I'm worried about bees and want to help save them
- [ ] To share the uncommon or rare species I find
- [ ] Recreational learning/Family activity
- [ ] Other: ____________________

10. **Do you have specialized training in bumble bees?**
Mark only one oval.

- Yes
- No
- Other: ____________

11. **Do you have any other wildlife identification skills? Please select the response that applies to you and include any additional information under "Other".**
Check all that apply.

- [ ] Yes - many
- [ ] Yes - some
- [ ] No - just bumble bees
- [ ] No - none
- [ ] Other: ____________

12. **Are you a member of any naturalist or wildlife based organisation or club? Please select all that apply and include any additional information under "Other".**
Check all that apply.

- [ ] Yes, active
- [ ] Yes, past
- [ ] No
- [ ] Other: ____________

Bumble Bee Watch - User Demographics

General demographic questions for Bumble Bee Watch users.

13. **What is your age? ***
Mark only one oval.

- Under 12
- 12 - 17
- 18 - 24
- 25 - 34
- 35 - 44
- 45 - 54
- 55 - 64
- 65 - 74
- 75 +

14. **Where do you live? (Province/State/Territory). Please select one from the drop-down list.** *

Mark only one oval.

- Alabama
- Alaska
- Alberta
- American Samoa
- Arizona
- Arkansas
- British Columbia
- California
- Colorado
- Connecticut
- Delware
- District of Columbia
- Florida
- Georgia
- Guam
- Hawaii
- Idaho
- Illinois
- Indiana
- Iowa
- Kansas
- Kentucky
- Louisiana
- Maine
- Manitoba
- Maryland
- Massachusetts
- Michigan
- Minnesota
- Mississippi
- Missouri
- Montana
- Nebraska
- Nevada

- New Brunswick
- New Hampshire
- New Jersey
- New Mexico
- New York
- Newfoundland and Labrador
- North Carolina
- North Dakota
- Northern Mariana Islands
- Northwest Territories
- Nova Scotia
- Nunavut
- Ohio
- Oklahoma
- Ontario
- Oregon
- Pennsylvania
- Prince Edward Island
- Puerto Rico
- Rhode Island
- Quebec
- Saskatchewan
- South Carolina
- South Dakota
- Tennessee
- Texas
- U.S. Virgin Islands
- Utah
- Vermont
- Virginia
- Washington
- West Virginia
- Wisconsin
- Wyoming
- Yukon

15. **Which of the following best describes the area in which you live? ***

Mark only one oval.

- Urban
- Suburban
- Rural

16. **What is your level of education? ***

Mark only one oval.

- Some high school
- High school diploma or equivalency
- Some college/university
- College graduate
- Undergraduate degree
- Master's degree
- Professional degree
- PhD
- Post-doctoral training
- Trade, technical, or vocational training
- Other: ____________________

Bumble Bee Watch - User Feedback

Tell us what you think!

17. **What can we improve for users of Bumble Bee Watch?**

18. **What do you like about using Bumble Bee Watch?**

Species Identification Difficulty (from photo)

Please only answer the question for your biogeographic region (East/West).

Species are to be ranked from 1 to 5, where 1 is considered easy to identify and 5 is considered difficult to identify from a photograph.

Easy - you are able to spot one or two characteristics that allow you to quickly identify the species without consulting any reference materials.
Medium - you spend time looking for key characteristics to help you identify the species, and may or may not consult reference materials.
Difficult - you spend a considerable amount of time searching for key characteristics and consult reference materials to help identify the species.

19. **EASTERN REGION ONLY. (For the Western region, please skip to the following question). Please identify the level of difficulty in identifying the following species from a photograph; 1 is easy and 5 is difficult.**

Mark only one oval per row.

	1 - Easy	2 - Somewhat Easy	3 - Medium	4 - Somewhat Difficult	5 - Difficult	6 - N/A
Rusty-patched Bumble Bee (affinis)						
Black and Gold Bumble Bee (auricomus)						
Two-spotted Bumble Bee (bimaculatus)						
Gypsy Cuckoo Bumble Bee (bohemicus)						
Northern Amber Bumble Bee (borealis)						
Lemon Cuckoo Bumble Bee (citrinus)						
Yellow Bumble Bee (fervidus)						
Fernald Cuckoo Bumble Bee (flavidus)						
Southern Plains Bumble Bee (fraternus)						
Frigid Bumble Bee (frigidus)						
Brown-belted Bumble Bee (griseocollis)						
Common Eastern Bumble Bee (impatiens)						
Indiscriminate Cuckoo Bumble Bee (insularis)						
High Country Bumble Bee (kirbiellus)						
Morrison Bumble Bee (morrisoni)						
Nevada Bumble Bee (nevadensis)						
American Bumble Bee (pensylvanicus)						
Confusing Bumble Bee (perplexus)						
Red-belted Bumble Bee (rufocinctus)						
Sanderson Bumble Bee (sandersoni)						
Tri-coloured Bumble Bee (ternarius)						
Yellow-banded Bumble Bee (terricola)						

	1 - Easy	2 - Somewhat Easy	3 - Medium	4 - Somewhat Difficult	5 - Difficult	6 - N/A
Half-black Bumble Bee (vagans)						
Variable Cuckoo Bumble Bee (variabilis)						

20. **WESTERN REGION ONLY. (For the Eastern region, please return to the previous question). Please identify the level of difficulty in identifying the following species from a photograph; 1 is easy and 5 is difficult.**

Mark only one oval per row.

	1 - Easiest	2 - Easy	3 - Medium	4 - Difficult	5 - Most Difficult	6 - N/A
White-shouldered Bumble Bee (appositus)						
Black and Gold Bumble Bee (auricomus)						
Gypsy Cuckoo Bumble Bee (bohemicus)						
Two Form Bumble Bee (bifarius)						
Northern Amber Bumble Bee (borealis)						
Obscure Bumble Bee (caliginosus)						
Central Bumble Bee (centralis)						
Lemon Cuckoo Bumble Bee (citrinus)						
Crotch Bumble Bee (crotchii)						
Cryptic Bumble Bee (cryptarum)						
Yellow Bumble Bee (fervidus)						
Yellow-headed Bumble Bee (flavifrons)						
Franklin Bumble Bee (franklini)						
Southern Plains Bumble Bee (fraternus)						
Frigid Bumble Bee (frigidus)						
Brown-belted Bumble Bee (griseocollis)						
Common Eastern Bumble Bee (impatiens)						
Indiscriminate Cuckoo Bumble Bee (insularis)						
White-tailed Bumbled Bee (jonellus)						
Black-tailed Bumble Bee (melanopygus)						
Fuzzy-horned Bumble Bee (mixtus)						
Morrison Bumble Bee (morrisoni)						
Nevada Bumble Bee (nevadensis)						
Western Bumble Bee (occidentalis)						
American Bumble Bee (pensylvanicus)						

	1 - Easiest	2 - Easy	3 - Medium	4 - Difficult	5 - Most Difficult	6 - N/A
Confusing Bumble Bee (perplexus)						
Red-belted Bumble Bee (rufocinctus)						
Sanderson Bumble Bee (sandersoni)						
Suckley Cuckoo Bumble Bee (suckleyi)						
Forest Bumble Bee (sylvicola)						
Tri-coloured Bumble Bee (ternarius)						
Half-black Bumble Bee (vagans)						
Van Dyke Bumble Bee (vandykei)						
Vosnesensky Bumble Bee (vosnesenski)						

Final Thoughts

21. **Please feel free to leave any additional comments about this survey or about Bumble Bee Watch in general.**

Thank you!

Thanks very much for taking the time to participate in the Bumble Bee Watch User Survey!

Powered by Google Forms
