# Supplementary material for: Community science participants gain environmental awareness and contribute high quality data but improvements are needed: insights from Bumble Bee Watch
Source: PeerJ. 2020 May 12;8:e9141. doi: 10.7717/peerj.9141 (PMC7227640; doi:10.7717/peerj.9141)
Supplement: Article S2 [file peerj-08-9141-s005.pdf]

Bumble Bee Watch - Expert Survey

You have been selected as an individual with expertise in bumble bee identification.

We are attempting to build an understanding of the application of citizen science in biodiversity conservation. As Bumble Bee Watch.org has generated a remarkable database of bumble bee records, researchers would like to know more about the website’s users. Learning about participants demographics, motives, and confidence with bumble bee identification is highly useful for researchers who wish to understand the possibly wide-ranging application of citizen science in the future.

As a complement to a separate survey of Bumble Bee Watch users, we are asking for your participation in our expert survey. (If you are interested in taking the user survey, please follow the link at the end of this survey). We are interested in your feedback regarding the ease or difficulty of identifying various species in the eastern and/or western regions of North America, whether you have verified submissions for us in the past or if you are interested in verifying submissions for us in the future, and your thoughts on the kinds of research questions that we might ask with the data collected through Bumble Bee Watch.

* Required

Informed Consent to Participate in Research

Please read the informed consent form and indicate your consent below.

1. **By checking the box below you indicate that you have read the form, below, and consent to participate in the BumbleBeeWatch.org survey conducted by York University research team.** *

   Check all that apply.

   ☐ I consent.

Additional Consent

Consent to waive anonymity

2. **Please indicate your consent below to the use of your name in the publications arising from this research.** *

   Mark only one oval.

   ◯ Waive anonymity

   ◯ Remain anonymous

Informed Consent Form

Date: September 26, 2017

Study Name: Assessing the Quality of BumbleBeeWatch.org Data

Researcher name:
Faculty of Environmental Studies
York University
Health, Nursing and Environmental Studies Building
4700 Keele St, Toronto, ON
M3J 1P3

Principal Investigator: Dr. Sheila Colla, PhD, srcolla@yorku.ca
Student Researchers: Shelby Gibson, MES candidate, sgibs94@yorku.ca, Madeleine Lavin, MES candidate, mlavin@yorku.ca, Victoria MacPhail, PhD candidate, vmacphail@gmail.com

Purpose of the Research:

You are being asked to participate in a short (5-10 min) survey because you are a user of BumbleBeeWatch.org. York University is a partner on the BumbleBeeWatch program, and are interested in assessing the quality of data collected through citizen science programs, such as BumbleBeeWatch. Part of this research requires an understanding of who is submitting observations to the database, as well as their motives for doing so. We do not foresee any risks or discomfort from your participation in the research. The benefit of participation is that the participant is able to contribute to a greater understanding of the application of citizen science research in biodiversity conservation. There is no requirement to complete the survey, and once a participant has begun the survey they reserve the right to quit at any point. The survey responses remain anonymous unless permission is given otherwise. As this survey is being administered online, we are relying on this informed consent letter (as opposed to a signature form) to fulfill the duty of informed consent. Further, the data collected may be used in a peer-reviewed publication and conference presentations in the future.

Voluntary Participation and Withdrawal:

Your participation in the study is completely voluntary and you may choose to stop participating at any time. Your decision not to volunteer, to stop participating, or to refuse to answer particular questions will not influence the nature of the ongoing relationship you may have with the researchers or study staff, or the nature of your relationship with York University either now, or in the future. In the event you withdraw from the study, all associated data collected will be immediately destroyed wherever possible. Should you wish to withdraw after the study, you will have the option to also withdraw your data up until the analysis is complete.

Confidentiality:

The method of documentation is through an online Google Survey, which will have an

research ethics | YORK UNIVERSITÉ UNIVERSITY

Office of Research Ethics York University Kaneff Tower, Fifth Floor – 4700 Keele Street, Toronto, Ontario, Canada, M3J 1P3 ore@yorku.ca research.info.yorku.ca

option to export the data collected in to an Excel spreadsheet. The data will be stored on a locked laptop for two years. After this, it may be added to an online data repository. This will allow for the data to be used in future research which may look at citizen science in biodiversity conservation.

Unless you choose otherwise, all information you supply during the research will be held in confidence and unless you specifically indicate your consent, your name will not appear in any report or publication of the research. Your data will be safely stored on a locked laptop, and only the research team will have access to this information. By September 2019, the data will either be destroyed or added to an online data repository. Confidentiality will be provided to the fullest extent possible by law.

The data collected in this research project may be used – in an anonymized form - by members of the research team in subsequent research investigations exploring similar lines of inquiry. Such projects will still undergo ethics review by the HPRC, our institutional REB. Any secondary use of anonymized data by the research team will be treated with the same degree of confidentiality and anonymity as in the original research project. If data is added to a repository, it will be done so in an anonymized manner.

The researcher(s) acknowledge that the host of the online survey (e.g., Qualtrix, Survey Monkey etc.) may automatically collect participant data without their knowledge (i.e., IP addresses.) Although this information may be provided or made accessible to the researchers, it will not be used or saved without participant's consent on the researcher's system. Further, "Because this project employs e-based collection techniques, data may be subject to access by third parties as a result of various security legislation now in place in many countries and thus *the confidentiality and privacy of data cannot be guaranteed during web-based transmission.*

Questions About the Research? If you have questions about the research in general or about your role in the study, please feel free to contact me at sgibs94@yorku.ca , or my supervisor, Dr. Sheila Colla at srcolla@yorku.ca. You may also contact the Graduate Program in Faculty of Environmental Studies at York University, (416)-736-5252.

This research has received ethics review and approval by the Delegated Ethics Review Committee, which is delegated authority to review research ethics protocols by the Human Participants Review Sub-Committee, York University's Ethics Review Board, and conforms to the standards of the Canadian Tri-Council Research Ethics guidelines. If you have any questions about this process, or about your rights as a participant in the study, please contact the Sr. Manager & Policy Advisor for the Office of Research Ethics, 5th Floor, Kaneff Tower, York University (telephone 416-736-5914 or e-mail ore@yorku.ca).

Legal Rights and Signatures:

I_________________________consent to participate in the BumbleBeeWatch.org survey conducted by York University research team. I have understood the nature of this project and wish to participate. I am not waiving any of my legal rights by signing this form. My signature

Office of Research Ethics
York University
Kaneff Tower, Fifth Floor – 4700 Keele Street,
Toronto, Ontario, Canada, M3J 1P3
ore@yorku.ca
research.info.yorku.ca

below indicates my consent.

Signature ______________________ **Date** ______________________
Participant

______________________ November 28, 2017
Principal Investigator

Additional consent:

1. **Consent to waive anonymity**

I, ______________________ consent to the use of my name in the publications arising from this research.

Signature ______________________ **Date** ______________________
Participant Name:

Introductory Questions

Please state your name and biogeographic region.

3. **Please state your name:** *

4. **Please select your biogeographic region:** *

Mark only one oval.

- ( ) Eastern Region - United States: East of the Mississippi | Canada: East of Ontario (including Ontario)
- ( ) Western Region - United States: West of the Mississippi | Canada: West of Manitoba (including Manitoba)

Bumble Bee Watch - Participation/Verification

5. **Have you submitted photos to Bumble Bee Watch?** *

Mark only one oval.

- ( ) Yes
- ( ) No

6. **Have you verified submissions to Bumble Bee Watch in the past?** *

Mark only one oval.

- ( ) Yes
- ( ) No

7. **If you have not verified submissions, would you be interested in verifying submissions for us in the future? (If no, please indicate why under "Other").**

Check all that apply.

- [ ] Yes
- [ ] No
- [ ] Other: ______________________

Bumble Bee Watch - Expert Research

We would like to know about your research! (Optional)

8. **Are you involved in any citizen science projects? If yes, please feel free to include details under "Other".**

Check all that apply.

- [ ] Yes
- [ ] No
- [ ] Other: ______________________

9. **In your research, have you used data from Bumble Bee Watch? If yes, please include details under "Other".**

Check all that apply.

- [ ] Yes
- [ ] No
- [ ] No but I would like to in the future
- [ ] Other: ______________________

Bumble Bee Watch - Research Questions

We are interested in hearing from you about the kinds of research questions that might come out of the citizen science data collected through the Bumble Bee Watch project.

10. **What kinds of research questions do you think the data collected through Bumble Bee Watch might be useful for?**

Bumble Bee Watch - Project Feedback

If you have participated in Bumble Bee Watch by submitting photos, verifying submissions, or using data, we'd appreciate your feedback! (Optional)

11. **Comments on submitting photos to Bumble Bee Watch:**

12. **Comments on verifying submissions on Bumble Bee Watch:**

13. **Comments on using data from Bumble Bee Watch:**

Species Identification Difficulty (from photo)

Please note that the following species lists are separated by biogeographic region (East/West). Please feel free to rank the ease or difficulty for the eastern and/or western biogeographic regions.

Species are to be ranked from 1 to 5, where 1 is considered easy to identify and 5 is considered difficult to identify from a photograph.

Easy - you are able to spot one or two characteristics that allow you to quickly identify the species without consulting any reference materials.
Medium - you spend time looking for key characteristics to help you identify the species, and may or may not consult reference materials.
Difficult - you spend a considerable amount of time searching for key characteristics and consult reference materials to help identify the species.

14. **EASTERN REGION ONLY. (For the Western region, please skip to the following question). Please identify the level of difficulty in identifying the following species from a photograph; 1 is easy and 5 is difficult.**

Mark only one oval per row.

	1 - Easy	2 - Somewhat Easy	3 - Medium	4 - Somewhat Difficult	5 - Difficult	6 - N/A
Rusty-patched Bumble Bee (affinis)	◯	◯	◯	◯	◯	◯
Black and Gold Bumble Bee (auricomus)	◯	◯	◯	◯	◯	◯
Two-spotted Bumble Bee (bimaculatus)	◯	◯	◯	◯	◯	◯
Gypsy Cuckoo Bumble Bee (bohemicus)	◯	◯	◯	◯	◯	◯
Northern Amber Bumble Bee (borealis)	◯	◯	◯	◯	◯	◯
Lemon Cuckoo Bumble Bee (citrinus)	◯	◯	◯	◯	◯	◯
Yellow Bumble Bee (fervidus)	◯	◯	◯	◯	◯	◯
Fernald Cuckoo Bumble Bee (flavidus)	◯	◯	◯	◯	◯	◯
Southern Plains Bumble Bee (fraternus)	◯	◯	◯	◯	◯	◯
Frigid Bumble Bee (frigidus)	◯	◯	◯	◯	◯	◯
Brown-belted Bumble Bee (griseocollis)	◯	◯	◯	◯	◯	◯
Common Eastern Bumble Bee (impatiens)	◯	◯	◯	◯	◯	◯
Indiscriminate Cuckoo Bumble Bee (insularis)	◯	◯	◯	◯	◯	◯
High Country Bumble Bee (kirbiellus)	◯	◯	◯	◯	◯	◯
Morrison Bumble Bee (morrisoni)	◯	◯	◯	◯	◯	◯
Nevada Bumble Bee (nevadensis)	◯	◯	◯	◯	◯	◯
American Bumble Bee (pensylvanicus)	◯	◯	◯	◯	◯	◯
Confusing Bumble Bee (perplexus)	◯	◯	◯	◯	◯	◯
Red-belted Bumble Bee (rufocinctus)	◯	◯	◯	◯	◯	◯
Sanderson Bumble Bee (sandersoni)	◯	◯	◯	◯	◯	◯
Tri-coloured Bumble Bee (ternarius)	◯	◯	◯	◯	◯	◯
Yellow-banded Bumble Bee (terricola)	◯	◯	◯	◯	◯	◯

	1 - Easy	2 - Somewhat Easy	3 - Medium	4 - Somewhat Difficult	5 - Difficult	6 - N/A
Half-black Bumble Bee (vagans)						
Variable Cuckoo Bumble Bee (variabilis)						

15. **WESTERN REGION ONLY. (For the Eastern region, please return to the previous question). Please identify the level of difficulty in identifying the following species from a photograph; 1 is easy and 5 is difficult.**

Mark only one oval per row.

	1 - Easiest	2 - Easy	3 - Medium	4 - Difficult	5 - Most Difficult	6 - N/A
White-shouldered Bumble Bee (appositus)						
Black and Gold Bumble Bee (auricomus)						
Gypsy Cuckoo Bumble Bee (bohemicus)						
Two Form Bumble Bee (bifarius)						
Northern Amber Bumble Bee (borealis)						
Obscure Bumble Bee (caliginosus)						
Central Bumble Bee (centralis)						
Lemon Cuckoo Bumble Bee (citrinus)us						
Crotch Bumble Bee (crotchii)						
Cryptic Bumble Bee (cryptarum)						
Yellow Bumble Bee (fervidus)						
Yellow-headed Bumble Bee (flavifrons)						
Franklin Bumble Bee (franklini)						
Southern Plains Bumble Bee (fraternus)						
Frigid Bumble Bee (frigidus)						
Brown-belted Bumble Bee (griseocollis)						
Common Eastern Bumble Bee (impatiens)						
Indiscriminate Cuckoo Bumble Bee (insularis)						
White-tailed Bumbled Bee (jonellus)						
Black-tailed Bumble Bee (melanopygus)						
Fuzzy-horned Bumble Bee (mixtus)						
Morrison Bumble Bee (morrisoni)						
Nevada Bumble Bee (nevadensis)						
Western Bumble Bee (occidentalis)						
American Bumble Bee (pensylvanicus)						

	1 - Easiest	2 - Easy	3 - Medium	4 - Difficult	5 - Most Difficult	6 - N/A
Confusing Bumble Bee (perplexus)						
Red-belted Bumble Bee (rufocinctus)						
Sanderson Bumble Bee (sandersoni)						
Suckley Cuckoo Bumble Bee (suckleyi)						
Forest Bumble Bee (sylvicola)						
Tri-coloured Bumble Bee (ternarius)						
Half-black Bumble Bee (vagans)						
Van Dyke Bumble Bee (vandykei)						
Vosnesensky Bumble Bee (vosnesenski)						

Bumble Bee Watch - User Survey

Please click the following link if you'd also like to participate in the Bumble Bee Watch User Survey:
https://goo.gl/forms/FYlxAn7Jgt4Pca4o2

Final Thoughts

16. **Please feel free to leave any additional comments about this survey or about Bumble Bee Watch in general.**

Thank you!

Thanks very much for taking the time to participate in the Bumble Bee Watch Expert Survey!

Powered by
Google Forms
