# Supplementary material for: Community science participants gain environmental awareness and contribute high quality data but improvements are needed: insights from Bumble Bee Watch
Source: PeerJ. 2020 May 12;8:e9141. doi: 10.7717/peerj.9141 (PMC7227640; doi:10.7717/peerj.9141)
Supplement: Table S1 — Significance levels were adjusted to control False Discovery Rates (FDRs) using the Benjamini-Hochberg procedure. [file peerj-08-9141-s007.docx]

Table S1. Results for a series of Kruskal-Wallis test statistics for the comparisons of user survey respondents’ motivations by age of respondent, number of years participating in Bumble Bee Watch, or by area they live (urban/suburban/rural). Significance levels were adjusted to control False Discovery Rates (FDRs) using the Benjamini-Hochberg procedure.

| Grouping Variable | Motivation | Kruskal-Wallis H Statistic | df | *p*-value | FDR-adjusted *p*-value |
| --- | --- | --- | --- | --- | --- |
| Respondent Age | To contribute to scientific data collection | 6.01 | 7 | 0.539 | 0.774 |
| Respondent Age | I want to learn what species are on my property | 9.471 | 7 | 0.221 | 0.553 |
| Respondent Age | I want to learn how to identify the biodiversity in my region | 2.456 | 7 | 0.93 | 0.999 |
| Respondent Age | Participation in special events (e.g. Great Canadian Bumble Bee Count or a Bioblitz) | 0.653 | 7 | 0.999 | 0.999 |
| Respondent Age | I have a personal interest in bumble bees | 11.155 | 7 | 0.132 | 0.493 |
| Respondent Age | I'm worried about bees and want to help save them | 10.793 | 7 | 0.148 | 0.493 |
| Respondent Age | To share the uncommon or rare species I find | 13.058 | 7 | 0.071 | 0.493 |
| Respondent Age | The preservation of ecological diversity | 3.75 | 7 | 0.808 | 0.999 |
| Respondent Age | Recreational learning/Family activity | 6.572 | 7 | 0.475 | 0.774 |
|  |  |  |  |  |  |
| Years Participated | To contribute to scientific data collection | 9.414 | 4 | 0.052 | 0.173 |
| Years Participated | I want to learn what species are on my property | 1.501 | 4 | 0.826 | 0.899 |
| Years Participated | I want to learn how to identify the biodiversity in my region | 1.07 | 4 | 0.899 | 0.899 |
| Years Participated | Participation in special events (e.g. Great Canadian Bumble Bee Count or a Bioblitz) | 3.417 | 4 | 0.491 | 0.739 |
| Years Participated | I have a personal interest in bumble bees | 6.755 | 4 | 0.149 | 0.373 |
| Years Participated | I'm worried about bees and want to help save them | 4.831 | 4 | 0.305 | 0.610 |
| Years Participated | To share the uncommon or rare species I find | 10.846 | 4 | 0.028 | 0.140 |
| Years Participated | The preservation of ecological diversity | 3.222 | 4 | 0.521 | 0.739 |
| Years Participated | Recreational learning/Family activity | 2.804 | 4 | 0.591 | 0.739 |
|  |  |  |  |  |  |
| Area Live In | To contribute to scientific data collection | 0.041 | 2 | 0.98 | 0.980 |
| Area Live In | I want to learn what species are on my property | 9.036 | 2 | 0.011 | 0.090 |
| Area Live In | I want to learn how to identify the biodiversity in my region | 1.966 | 2 | 0.374 | 0.637 |
| Area Live In | Participation in special events (e.g. Great Canadian Bumble Bee Count or a Bioblitz) | 8.029 | 2 | 0.018 | 0.090 |
| Area Live In | I have a personal interest in bumble bees | 2.921 | 2 | 0.232 | 0.580 |
| Area Live In | I'm worried about bees and want to help save them | 0.758 | 2 | 0.685 | 0.761 |
| Area Live In | To share the uncommon or rare species I find | 6.894 | 2 | 0.032 | 0.107 |
| Area Live In | The preservation of ecological diversity | 1.923 | 2 | 0.382 | 0.637 |
| Area Live In | Recreational learning/Family activity | 1.36 | 2 | 0.507 | 0.724 |
