# Supplementary material for: Community science participants gain environmental awareness and contribute high quality data but improvements are needed: insights from Bumble Bee Watch
Source: PeerJ. 2020 May 12;8:e9141. doi: 10.7717/peerj.9141 (PMC7227640; doi:10.7717/peerj.9141)
Supplement: Table S2 — Note that respondents could make multiple suggestions so the total of all categories do not add up to 100%. Free-form responses were initially coded into one of 29 categories by three individuals, and then further collapsed into these six categories. [file peerj-08-9141-s008.docx]

Table S2. Categories where user survey respondents (n=210) suggested improvements could be made to the Bumble Bee Watch program. Note that respondents could make multiple suggestions so the total of all categories do not add up to 100%. Free-form responses were initially coded into one of 29 categories by three individuals, and then further collapsed into these six categories.

| **Suggested Improvement Areas** | **Number of Respondents** | **Percent of Respondents** |
| --- | --- | --- |
| Happy - nothing to improve, can't think of anything | 33 | 15.7 |
| Feedback to users - faster identifications, more details on their submissions/identifications | 58 | 27.6 |
| IT related - improvement of app and website functionality, submission process (including showing real photos in addition to the diagrams) | 88 | 41.9 |
| Tools and resources - program usage tips, identification guides, local species lists, tutorials/workshops, etc. (for bees and plants) | 40 | 19.0 |
| Increased communication - results, reports, general feedback, etc. | 24 | 11.4 |
| Other ideas, comments | 20 | 9.5 |
