# Supplementary material for: Community science participants gain environmental awareness and contribute high quality data but improvements are needed: insights from Bumble Bee Watch
Source: PeerJ. 2020 May 12;8:e9141. doi: 10.7717/peerj.9141 (PMC7227640; doi:10.7717/peerj.9141)
Supplement: Table S3 — There were 342 survey respondents and 23,017 records in the Bumble Bee Watch database as of February 28, 2018, covering both the United States and Canada. The United States population during the 2015–2017 period was 321,004,407 (excluding Puerto Rico) (United States Census Bureau, 2017a) , and the Canadian population in 2016 was 35,151,728 (Statistics Canada, 2017b) , for a combined population of 356,156,135. [file peerj-08-9141-s009.docx]

Table S3: A comparison of the relative percentage of user survey respondents, database records, and combined population per United States state or territory and Canadian province or territory (“jurisdiction”). There were 342 survey respondents and 23,017 records in the Bumble Bee Watch database as of February 28, 2018, covering both the United States and Canada. The United States population during the 2015-2017 period was 321,004,407 (excluding Puerto Rico)(United States Census Bureau, 2017a), and the Canadian population in 2016 was 35,151,728 (Statistics Canada, 2017b), for a combined population of 356,156,135.

| Federal Jurisdiction | Percent of User Survey Respondents | Percent of Records in Database | Percent of Combined National Populations |
| --- | --- | --- | --- |
| Alabama | 0.88 | 0.47 | 1.36 |
| Alaska | 0.00 | 0.25 | 0.21 |
| Alberta | 3.80 | 4.99 | 1.14 |
| Arizona | 0.58 | 0.16 | 1.91 |
| Arkansas | 0.29 | 0.11 | 0.84 |
| British Columbia | 4.97 | 5.21 | 1.31 |
| California | 2.92 | 2.87 | 10.95 |
| Colorado | 4.39 | 1.72 | 1.53 |
| Connecticut | 0.58 | 0.10 | 1.01 |
| Delaware | 0.00 | 0.17 | 0.26 |
| District of Columbia | 0.29 | 0.03 | 0.19 |
| Florida | 1.75 | 0.51 | 5.69 |
| Georgia | 0.29 | 0.36 | 2.86 |
| Hawaii | 0.00 | 0.00 | 0.40 |
| Idaho | 1.75 | 1.48 | 0.47 |
| Illinois | 1.46 | 1.26 | 3.61 |
| Indiana | 0.88 | 0.68 | 1.86 |
| Iowa | 0.88 | 0.49 | 0.88 |
| Kansas | 0.29 | 0.17 | 0.82 |
| Kentucky | 0.00 | 0.22 | 1.24 |
| Louisiana | 0.00 | 0.05 | 1.31 |
| Maine | 1.46 | 2.36 | 0.37 |
| Manitoba | 1.17 | 1.89 | 0.36 |
| Maryland | 2.05 | 0.73 | 1.68 |
| Massachusetts | 1.17 | 0.85 | 1.91 |
| Michigan | 2.63 | 2.35 | 2.79 |
| Minnesota | 7.60 | 6.05 | 1.54 |
| Mississippi | 0.00 | 0.06 | 0.84 |
| Missouri | 1.75 | 0.58 | 1.71 |
| Montana | 0.29 | 1.24 | 0.29 |
| Nebraska | 0.29 | 0.49 | 0.53 |
| Nevada | 0.00 | 0.25 | 0.81 |
| New Brunswick | 0.29 | 0.66 | 0.21 |
| New Hampshire | 0.29 | 0.53 | 0.37 |
| New Jersey | 0.58 | 0.40 | 2.52 |
| New Mexico | 0.58 | 0.16 | 0.59 |
| New York | 2.63 | 1.49 | 5.56 |
| Newfoundland and Labrador | 0.00 | 0.31 | 0.15 |
| North Carolina | 0.58 | 0.87 | 2.82 |
| North Dakota | 0.00 | 0.15 | 0.21 |
| Northwest Territories | 0.29 | 0.13 | 0.01 |
| Nova Scotia | 1.75 | 1.63 | 0.26 |
| Nunavut | 0.00 | 0.03 | 0.01 |
| Ohio | 3.22 | 1.99 | 3.26 |
| Oklahoma | 0.00 | 0.33 | 1.09 |
| Ontario | 18.13 | 26.50 | 3.78 |
| Oregon | 5.85 | 5.73 | 1.13 |
| Pennsylvania | 1.46 | 1.33 | 3.59 |
| Prince Edward Island | 0.29 | 0.19 | 0.04 |
| Quebec | 1.46 | 1.04 | 2.29 |
| Rhode Island | 0.00 | 0.06 | 0.30 |
| Saskatchewan | 3.22 | 1.92 | 0.31 |
| South Carolina | 0.00 | 0.33 | 1.37 |
| South Dakota | 0.29 | 0.21 | 0.24 |
| Tennessee | 1.46 | 0.40 | 1.85 |
| Texas | 1.17 | 1.58 | 7.70 |
| Utah | 0.88 | 0.40 | 0.84 |
| Vermont | 1.17 | 0.85 | 0.18 |
| Virginia | 1.75 | 1.02 | 2.35 |
| Washington | 4.39 | 7.07 | 2.01 |
| West Virginia | 0.00 | 0.08 | 0.52 |
| Wisconsin | 3.51 | 3.95 | 1.62 |
| Wyoming | 0.29 | 0.49 | 0.16 |
| Yukon | 0.00 | 0.05 | 0.01 |
