# Supplementary material for: Community science participants gain environmental awareness and contribute high quality data but improvements are needed: insights from Bumble Bee Watch
Source: PeerJ. 2020 May 12;8:e9141. doi: 10.7717/peerj.9141 (PMC7227640; doi:10.7717/peerj.9141)
Supplement: Table S4 — Significance levels were adjusted to control False Discovery Rates (FDRs) using the Benjamini-Hochberg procedure. [file peerj-08-9141-s010.docx]

Table S4. Statistical output for a series of Dunn’s post-hoc tests comparing the total number of years the user survey respondents have participated in Bumble Bee Watch to their reported number (class) of submissions. Significance levels were adjusted to control False Discovery Rates (FDRs) using the Benjamini-Hochberg procedure.

| Years of Participation Comparisons | Test Statistic | *p*-value | FDR-adjusted *p*-value |
| --- | --- | --- | --- |
| year 0 & 1 | 9.04 | 0.49 | 0.49 |
| year 0 & 2 | -20.01 | 0.09 | 0.10 |
| year 0 & 3 | -63.96 | <0.001 | <0.001 |
| year 0 & 4 | -104.86 | <0.001 | <0.001 |
| year 1 & 2 | -29.05 | 0.02 | 0.02 |
| year 1 & 3 | -73.01 | <0.001 | <0.001 |
| year 1 & 4 | -113.90 | <0.001 | <0.001 |
| year 2 & 3 | -43.96 | <0.001 | 0.001 |
| year 2 & 4 | -84.85 | <0.001 | <0.001 |
| year 3 & 4 | -40.90 | 0.04 | 0.054 |
